# A Cosmopolitan Argument for Temporary “Diagonal” Short-Term Surgical Missions as a Component of Surgical Systems Strengthening

**DOI:** 10.9745/GHSP-D-24-00046

**Published:** 2024-10-29

**Authors:** Gabriella Yael Hyman, Rashi Jhunjhunwala, Douglas W. Hanto

**Affiliations:** a University of the Witwatersrand Johannesburg School of Clinical Medicine, Johannesburg, South Africa.; b Beth Israel Deaconess Medical Center, Boston, MA, USA.; c Harvard Medical School Center for Bioethics, Boston, MA, USA.

## Abstract

We propose an argument for “diagonal” short-term surgical missions as a stop-gap component of global surgical systems strengthening based upon the political justice theory of moral cosmopolitanism

## Key Messages

- Short-term surgical missions (STSMs), which deploy surgical teams from high-resource settings to provide much-needed surgical care in underserved populations in low-resource settings, are a commonly used method of reducing the burden of surgical disease.
- To facilitate the much-needed paradigm shift in the way we conceptualize and conduct STSMs, we propose an argument for “diagonal” STSMs as a stop-gap component of global surgical systems strengthening based upon the political justice theory of moral cosmopolitanism.
- Hybrid “diagonal approaches” to STSMs have been proposed as the capacity-building model needed to provide immediate benefit and sustainable long-term impact for surgical health system improvement.
- Devising and implementing context-specific capacity-building interventions for the surgical care workforce and system-strengthening interventions requires significant community engagement to allow local stakeholders to guide the development and implementation of STSMs and ensure that programs are ethical, culturally sensitive, and sustainable.

## INTRODUCTION

The global burden of surgical disease remains largely unmet. While the issue of inequitable access to surgical care, particularly in resource-limited settings, is well described, effective solutions focused on health system expansion and strengthening remain inadequate. Geospatial barriers to care, inadequate surgical and anesthesia workforce density, limited resources and infrastructure, and competing public health priorities mean that health systems in low- and middle-income countries (LMICs) often are unable to meet the surgical needs of their populations.^1^

Much of the human and capital resources required to ameliorate inequitable surgical care provision and outcomes are concentrated in high-income countries (HICs)^1^. Many individuals and organizations in high-resourced settings feel compelled to act to reduce health inequity and pursue social justice. Short-term surgical missions (STSMs) are a commonly used method of reducing the burden of surgical disease. These missions deploy surgical teams from high-resource settings to provide much-needed surgical care in underserved populations. While this practice has existed for decades, more recently, practitioners and patients have raised concerns about the role STSMs play in global health neo-colonialism, the potential exploitation STSMs can perpetuate, and higher observed complication rates.^2^^,^^3^ This is largely due to a lack of sustainability, accountability, and ethical frameworks for undertaking STSMs.^2^

Normative theory and principles of distributive justice guide us in determining moral imperatives for how those engaged in partnerships to ameliorate health inequity should act and how to answer questions about resource allocation and “ownership.”^4^ In a chapter titled “How to maintain ethical standards of global surgery practice and partnerships,” Alayande et al. presented several ethical frameworks for consideration and application in engaging in global surgery partnerships and programs.^3^ These frameworks are especially necessary when considering the dynamics of political, economic, and institutional power within global governance structures. Yet, criticism within global health persists as to whether STSMs should ensue at all, who stands to benefit, and whether that benefit is proportional. A paradigm shift in the way we conceptualize and conduct STSMs is needed. We propose an argument for “diagonal” STSMs as a stop-gap component of global surgical systems strengthening based upon the political justice theory of moral cosmopolitanism.^5^

## THE DIAGONAL MODEL OF SHORT-TERM SURGICAL MISSIONS

Many STSM programs employ a “vertical approach” to health care delivery and lack the framework needed to ensure accountability for sustainable health systems strengthening. Vertical programs are usually disease focused, privately funded, and exist in parallel or outside local health systems.^3^^,^^6^ “Horizontal approaches” are committed to capacity-building and require long-term investments in health systems and infrastructure across domains.^6^ Where vertical programs focus on providing needed surgical services for defined populations, horizontal programs may focus on training and expanding the surgical care workforce or upscaling sustainable infrastructure. Hybrid “diagonal approaches” to STSMs have been proposed as the capacity-building model needed to provide immediate benefit and sustainable long-term impact for surgical health system improvement.^6^ In other words, while investing the time to build high-quality and resilient health systems, we can simultaneously help patients who are suffering from a lack of access to surgical care. Examples of vertical and horizontal interventions in global surgery, with STSMs presented as a diagonal “bridge,” are presented in the Figure. However, the asymmetric power dynamics inherent to global health partnerships render the ethics surrounding even diagonal STSMs potentially problematic.

**FIGURE:**
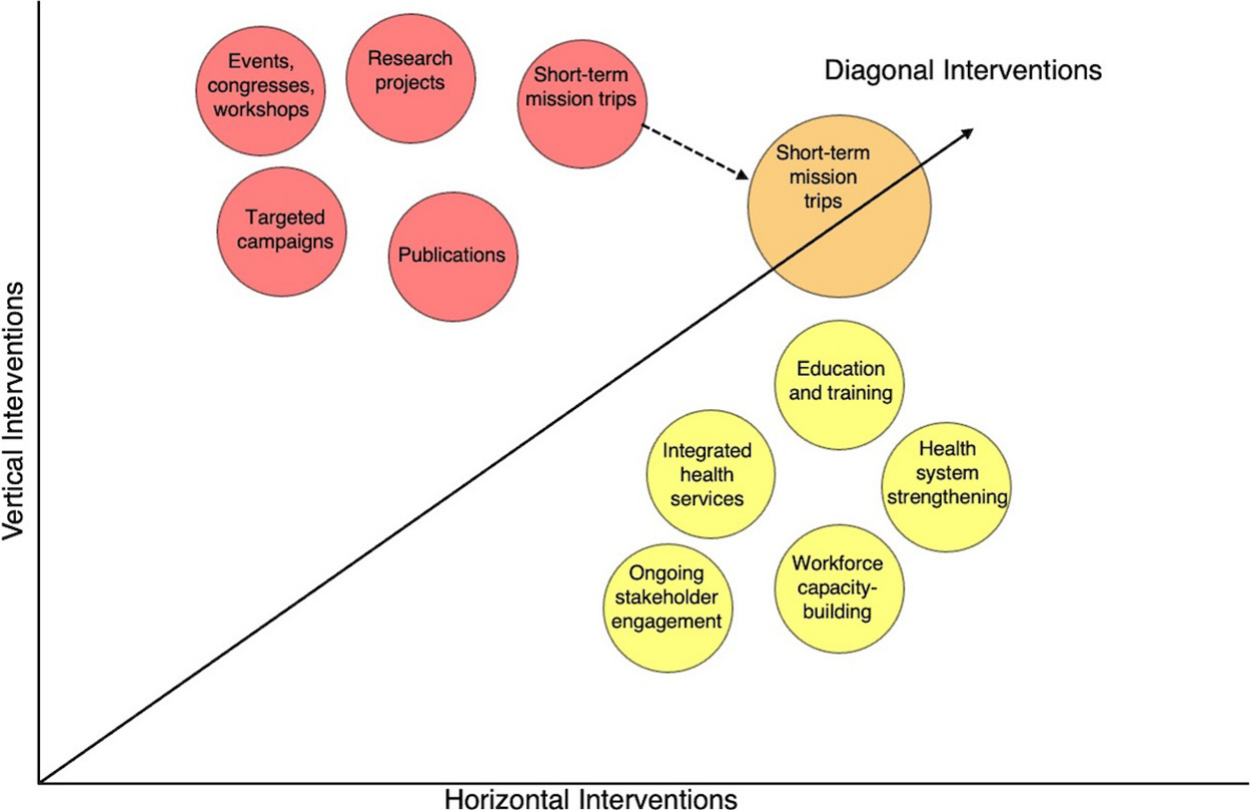
Examples of Vertical and Horizontal Interventions for Surgical Care Delivery^a^ ^a^ The axes are unweighted, and no scale or prioritization methods have been applied to the examples presented. When undertaken longitudinally and/or integrated into horizontal interventions, vertical interventions can transition into diagonal interventions.

While investing the time to build high-quality and resilient health systems, we can simultaneously help patients who are suffering from a lack of access to surgical care.

## COSMOPOLITANISM AND GLOBAL HEALTH CARE

There are numerous challenges to developing and implementing an ideal model for equitable redistribution of resources in under-resourced settings. Moral cosmopolitanism, which presupposes that political borders are less meaningful than our common humanity, dictates that little to no extra weighting be given to providing surgical care to compatriots in high-resourced settings purely due to geographical proximity.^4^ It suggests a needs-based approach to surgical resource allocation based on humanity’s moral responsibility toward humanity. In the modern era, where communities of providers and recipients transcend geographical boundaries and are often based on socioeconomic status or demographics, a cosmopolitan argument for the ethical obligation to provide equitable surgical care, regardless of state, location, or geopolitical factors, is pertinent. One has no control over the systemic, structural, and social circumstances into which one is born and which determine and perpetuate health inequalities.

In contrast, a statist approach—which prioritizes the needs of a political state and its citizens before allocating resources for humanitarian aid—disregards the effects of an increasingly globalized world.^4^ Cosmopolitan theory puts forth a moral obligation of individuals in positions of power and privilege to rectify unequal structures. The cross-sectoral disparities in wealth, resources, and education, many of which are a result of colonialism, continue to reinforce HICs’ (and individuals’) positions of dominance in health care. In fact, taking a zero-tolerance approach to STSMs or global health partnerships involving HICs may worsen the wealth gap and exacerbate social, economic, and technological disparities that further disadvantage surgical patients. Acknowledgment of these realities can justify and provide reason for the obligation of HIC actors to enact a sustainable pattern of redistribution in a way that promotes equity in access to care.

Within this framework, STSMs could be, prima facie, valid and valuable for achieving global distributive justice in the context of surgical health care. This then creates an expectation for STSMs to meet each system’s needs and provide goals for improving surgical care for the global community of patients and practitioners. Engagement with planning, execution, iteration, and adjustment to partners’ needs can fortify the ethical actions that support these activities.^3^

## STRUCTURING SHORT-TERM SURGICAL MISSIONS FOR ETHICAL AND SUSTAINABLE OUTCOMES

The way STSMs are carried out is important. Surgical capacity-building and systems strengthening take time. To act in a way that is ethical and sustainable, significant engagement with the community and investment are needed in multiple aspects of the surgical health system. The 2 most common components of the surgical system with the potential to benefit from STSMs are the surgical care workforce and patient case volume. Several demonstrated examples of how diagonal STSM models amalgamate targeted interventions and sustainability have been described.^7^ Common practices include “train-the-trainer” and missions focused on skills transfer rather than procedure provision alone. HIC surgical care providers may travel abroad to provide training while also addressing a need for essential and often specialized surgical procedures, while follow-ups are arranged in collaboration with local or overlapping multidisciplinary teams.^2^^,^^3^ With this model, STSMs have the potential to be integrated longitudinally into horizontal models for health systems strengthening. This can be achieved successfully, as Frenk et al. described, through the “adoption of competency-based curricula that are responsive to rapidly changing needs rather than being dominated by static coursework. Competencies should be adapted to local contexts and be determined by national stakeholders while harnessing global knowledge and experiences.”^8^ Devising and implementing context-specific training programs allows local stakeholders in LMICs to guide STSMs, particularly those involved in workforce training, and ensure that programs are both diagonal and service the country’s needs.

Additionally, STSMs facilitate the inflow of critical equipment and supplies particularly for hard-to-reach places. With a diagonal approach, a region’s logistics and supply chain operations can be strengthened through collaboration with STSM infrastructure.^9^ The implementation of Operation Smile’s programs is a successful example of an STSM functioning as a diagonal model. What began as a 501c3 organization focused on medical missions and cost-effective surgeries for craniofacial anomaly resulted in the development of national teams of volunteers along with investments into equipment, supplies, and infrastructure for ongoing surgical care.^6^^,^^10^ Another successful example is how Kids Operating Room (Kids OR) has contributed to sustainably building the surgical health care workforce. To complement work installing operating rooms for children’s surgery, Kids OR works with multiple partners to train pediatric surgical care providers while building operating rooms and providing much-needed surgeries.^11^

If diagonal STSMs persist as an interim measure to decreasing the burden of surgical disease in low-resourced settings, bidirectional partnership-building, professional development (of all parties), community impact, financing, cultural sensitivity, and sustainable coordination become vital.^3^^,^^12^ Through a systematic review, Leversedge et al. provided a summary and analysis of available guidelines (and their limitations) for conducting ethical STSMs. These guidelines provide a much-needed framework for the technical development and implementation of sustainable STSMs. Emphasis is placed on the whole spectrum of surgical care—including screening, rehabilitation, and allied health services. However, even this most recent set of guidelines was derived by consensus of largely HIC-based authors. “Rules of engagement” in STSMs, diagonal or otherwise, should be LMIC-led so that community priorities are heard.^3^ Accountability measures must be created and incentives for adherence maintained.^3^^,^^13^

“Rules of engagement” in STSMs, diagonal or otherwise, should be LMIC-led so that community priorities are heard.

## CONCLUSION

Assuming that all components of a sustainable, accountable, and comprehensive framework are applied, the question remains how STSMs, even in the context of an applied standardized framework, can be ethically justified. Adjusted models of STSMs, even in South-to-South partnerships, require careful consideration and review. The obligation to address the inequitable concentration of resources transcends borders and exists within an individual country, between sectors and industries, and among homogenous and heterogenous communities. The nuanced set of normative principles that strive for common good and social justice make room for diagonal STSMs in the context of global health—though each program must undergo rigorous scrutiny to ensure ethically just implementation and follow-up. The ideal STSM seeks to become redundant. The proof of significant progress would be globally self-sustaining and independent surgical systems that provide high-quality, safe, affordable, and timely surgical care where it did not exist before. The application of a cosmopolitan ethical framework allows for movement toward this goal.

## Notes

**Peer Reviewed**

**First Published Online:** October 1, 2024.

## References

[1] Meara JG, Leather AJM, Hagander L, et al. Global Surgery 2030: evidence and solutions for achieving health, welfare, and economic development. Lancet. 2015;386(9993):569–624. 10.1016/s0140-6736(15)60160-x. 25924834

[2] Hendriks TCC, Botman M, Rahmee CNS, et al. Impact of short-term reconstructive surgical missions: a systematic review. BMJ Glob Health. 2019;4(2):e001176. 10.1136/bmjgh-2018-001176. 31139438 PMC6509599

[3] Alayande BT, Riviello RR, Bekele A. How to maintain ethical standards of global surgery practice and partnerships. In: Hardy MA, Hochman BR, eds. *Global Surgery: How to Work and Teach in Low-and Middle-Income Countries.* Vol 1. Springer; 2023:21–37.

[4] Brown GW. Distributing who gets what and why: four normative approaches to global health. Glob Policy. 2012;3(3):292–302. 10.1111/j.1758-5899.2012.00180.x

[5] Bernstein AR. Moral cosmopolitanism. In: Chatterjee DK, ed. Encyclopedia of Global Justice. Springer; 2011:711–717.

[6] Patel PB, Hoyler M, Maine R, Hughes CD, Hagander L, Meara JG. An opportunity for diagonal development in global surgery: cleft lip and palate care in resource-limited settings. Plast Surg Int. 2012:892437. 10.1155/2012/892437. 23316355 PMC3539333

[7] Botman M, Hendriks TCC, Keetelaar AJ, et al. From short-term surgical missions towards sustainable partnerships. A survey among members of foreign teams. Int J Surg Open. 2021;28:63–69. 10.1016/j.ijso.2020.12.006

[8] Frenk J Prof, Chen L Dr, Bhutta ZA Prof, et al. Health professionals for a new century: transforming education to strengthen health systems in an interdependent world. Lancet. 2010;376(9756):1923–1958. 10.1016/s0140-6736(10)61854-5. 21112623

[9] Tracey P, Rajaratnam E, Varughese J, et al. Guidelines for short-term medical missions: perspectives from host countries. Global Health. 2022;18(1):19. 10.1186/s12992-022-00815-7. 35183205 PMC8857875

[10] Magee WP Jr., Vander Burg R, Hatcher KW. Cleft lip and palate as a cost-effective health care treatment in the developing world. World J Surg. 2010;34(3):2014. 10.1007/s00268-009-0333-7. 20063097

[11] Wood G, Wood N, Cunningham D, et al. Strengthening Surgical Systems: The Smartest Investment in Global Health. Global Report 2023. Kids Operating Room; 2023. Accessed September 11, 2024. https://issuu.com/kidsoperatingroom/docs/global_report_2023_sheets

[12] Leversedge C, McCullough M, Appiani LMC, Đình MP, Kamal RN, Shapiro LM. Capacity building during short-term surgical outreach trips: a review of what guidelines exist. World J Surg. 2023;47(1):50–60. 10.1007/s00268-022-06760-1. 36210361 PMC9726663

[13] Zitzman E, Berkley H, Jindal RM. Accountability in global surgery missions. BMJ Glob Health. 2018;3(6):e001025. 10.1136/bmjgh-2018-001025. 30687523 PMC6326286

